# Local government debt and labor income share: Evidence from China

**DOI:** 10.1371/journal.pone.0293494

**Published:** 2023-10-26

**Authors:** Yuanlin Wu, Cunzhi Tian, Lifang Li

**Affiliations:** School of Economics, Jinan University, Guangzhou, China; The University of Hong Kong, HONG KONG

## Abstract

This study employs a CES production function to construct a theoretical model of labor income share and uses a two-way fixed effects model to test the causal effects of local government debt (LGD) on the labor income share of enterprises. Local government debt governance policies are utilized as exogenous shocks, and a DID (Difference-in-Differences) model is applied for endogeneity testing. The results have passed a series of robustness checks. The findings suggest that LGD decreases the share of firms’ labor income. The mechanism analysis suggests that LGD lowers the labor remuneration of residents, the employment of labor in enterprises, and the size of bank loans mainly; while raising the cost of using funds in enterprises. Moreover, this negative effect is more apparent in non-state-owned enterprises, small and medium-sized enterprises, and enterprises with high financing constraints. This study presents new evidence on how the labor income share of enterprises is affected from the perspective by local governments in China. It has important implications for further deepening local government debt governance and achieving common prosperity.

## 1. Introduction

After China’s comprehensive victory in the fight against poverty, the principal social contradiction has been changed into the contrast between the people’s increasingly better living needs and the uneven and inadequate development. Therefore, the promotion and achievement of common prosperity are of direct relevance to the well-being of the people. The Chinese central government stresses that common prosperity is an inherent socialist demand and that it should stick to the people-oriented developing ideology and promote the commonwealth in high-quality development. However, since the 1990s, China’s labor income share has generally shown a downward trend, falling to 40% in 2007 and has remained at a low level for a prolonged period [1]. When the labor income share declines, it will widen the income gap between labor and other factors of production, which will affect the achievement of the goal of common prosperity. Therefore, gradually increasing the labor income share and optimizing the distribution of labor factors are essential components of deepening income distribution reform to achieve shared prosperity.

While the existing literature primarily emphasizes the impact of LGD on economic development and risk, there is a growing need for a deeper investigation into its effects on income distribution. Examining the distributional effects of local government debt is essential as income distribution holds a pivotal role in economic research, alongside economic growth. In the context of China’s emphasis on common prosperity, it becomes paramount in determining whether individuals have equitable access to the fruits of economic development [2]. Income distribution involves two key steps: initial distribution and redistribution. Income distribution involves two primary steps: initial distribution and redistribution. In the initial distribution step, factors of production, primarily capital and labor, are compensated in accordance with their respective contributions [3, 4]. Currently, there is a growing academic focus on the labor income share, also known as labor share, which represents the portion allocated to labor in the first step of income distribution [5]. This paper aims to investigate the distributional effects of LGD with a specific focus on labor income share.

Government investment spending has been essential in promoting economic growth and achieving shared prosperity. Government debt financing has provided vital support to promote the construction and improvement of critical economic projects and livelihood projects. It further strengthens the economy’s stability and stimulates economic development, thus promoting investment by enterprises and creating a ‘‘crowding-in effect” [6]. When the level of government debt is low, borrowing can revitalize idle funds, replenish financial resources, promote input and output, improve infrastructure, increase labor supply, and improve people’s livelihood projects, thus boosting economic growth. A specific level of government debt can play a pivotal role in fostering economic development, but excessive debt can give rise to significant issues. When government debt surpasses the equilibrium point, it triggers what is known as the ‘‘crowding-out effect” [7]. In particular, the response of unexpected shocks to small-scale Chinese firms may be different [8, 9]. This effect primarily occurs when resources, such as credit capital, are relatively fixed or limited. The expansion of government financing or investment, achieved through resource reallocation, competition, and other channels, can lead to a reduction in the scale of corporate investment and financing.

The rise in LGD may have the direct consequence of crowding out firms’ financing channels, raising their financing costs, and causing them to face the dilemma of external financing constraints. The uncertain exogenous shocks may lead to capital and labor shortages in the region, resulting in economic damage [10]. Related literatures have found that the expansion of LGD distorts the allocation of commercial bank funds, crowds corporate credit resources, and heightens corporate financing constraints [11]. Financing constraints are an important channel that influences firms to make business decisions [12]. For example, firms maintain certain financial ratios that affect not only their stock returns, but also firm performance [13, 14]. Firms facing financing constraints often have limited access to working capital through credit channels. Consequently, they tend to rely on internal savings for financing, which can lead to reduced labor employment or a decrease in labor factor prices, thereby negatively impacting the labor share [15]. The variation in the labor share is not only linked to the price of the labor factor utilized by the firm but also to the price of the capital factor used by the firm. Excessive government debt issuance can lead to a decline in treasury bond prices and a rise in interest rates, increasing the cost of corporate debt financing. Financial institutions may demand higher returns on debt from firms, further elevating financing costs. Given the safety and liquidity of government bonds, investors often prefer them over corporate bonds, compelling firms to offer higher returns on debt to attract investors, consequently increasing the cost of financing. Additionally, the value of the elasticity of substitution between firms’ capital and labor factors is closely related to changes in labor income [16, 17]. However, the relationship between local government debt and labor income shares has yet to be discussed in the available literature, so the aim of this paper is to fill the gap in such research.

Establishing a causal relationship between LGD and labor income share requires rigorous economic analysis, particularly microeconomic modeling of individual decision-making. This entails building a theoretical framework that elucidates the connection between LGD, the labor force, and the mechanism through which LGD impacts firms’ labor income share. This paper finds that LGD significantly reduces the labor income share within corporations. However, this effect is mitigated when LGD is effectively managed. The empirical results of this research have undergone rigorous testing, including endogeneity tests and a series of robustness tests, all of which validate the findings. The mechanism analysis shows that local government debt mainly reduces the labor share by reducing the labor remuneration of residents, the labor demand of enterprises and the size of bank loans, and increases the cost of capital use. There is heterogeneity in the policy responses of different groups to government [18]. The heterogeneity analysis reveals that this negative effect is more significant among non-state-owned firms, smaller enterprises, and those facing higher financing constraints. Overall, this research provides valuable insights into the intricate relationship between LGD and labor income share, offering a comprehensive analysis that encompasses theory, empirical analysis, and detailed mechanisms.

Compared with the previous literature, the main contributions of the paper are centered on the points below: First, the economic consequences of LGD expansion are explored from the perspective of micro-firms, complementing the research on LGD’s impact on firms’ labor income shares and providing new theoretical and empirical support regarding the impact of LGD on firms. Second, this study provides a comprehensive and in-depth exploration in assessing the micro-mechanism of the impact of LGD on the labor share. The analysis is not only from the perspective of average employee wages but also includes detailed analysis from multiple perspectives, such as labor hiring, enterprise productivity, and the cost of capital use. The comprehensive analysis reveals how local government debt affects the distribution of labor income within firms, helping us to understand this complex relationship more comprehensively. Finally, this study is conducted in the context of China’s advocacy of common wealth. Focusing on the micro-firm level, it is argued that LGD governance can mitigate the crowding-out effect on firms’ labor income share. This finding provides a deeper understanding that can help promote economic construction and realize the goal of common wealth.

This paper is organized as follows for the rest of the paper. Section 2 describes LGD in the Chinese institutional context. Section 3 constructs a theoretical model of labor income share based on which the impact of LGD is introduced. Section 4 presents the empirical data and constructs the main variables. Section 5 empirically analyzes the results and robustness test of local government debt affecting firms’ labor income share. Section 6 further analyzes the mechanism and heterogeneity analysis of local government debt affecting labor income share. Section 7 is the conclusion and recommendation part.

## 2. Institutional background

### 2.1 Emergence and characterization of local government financing vehicles

Since the implementation of China’s tax-sharing reform, local governments have grappled with the challenge of a disconnect between their authority, expenditure responsibilities, and available financial resources. This predicament has resulted in a significant funding gap, particularly in the realm of economic development, with infrastructure construction being a notable example [19]. In response to this situation, local governments have established numerous state-owned investment entities, commonly referred to as local government financing vehicles (LGFVs) [20]. These LGFVs serve as proxies for local governments in infrastructure investments and have the capacity to issue bonds or secure loans from financial institutions to mobilize funds.

Especially after the outbreak of the global financial crisis in 2008, in order to cope with the global financial crisis, the Chinese government initiated a substantial economic stimulus package and lifted the restriction on local governments to raise debts. Debt financing emerged as the primary and swiftest method for boosting economic growth because debt-financed policy measures had a more direct impact on economic growth compared to other approaches. Consequently, local governments have set up LGFVs for this purpose. LGFVs are state-owned enterprises (SOEs) owned by local governments, primarily established to secure funds for public expenditures [21].

In the credit market, enterprises find themselves at a distinct disadvantage when competing with local governments, resulting in a disproportionate flow of regional credit resources towards the government sector. Several factors contribute to this imbalance. Firstly, LGFVs are established and controlled by local governments, with key personnel appointed by these governments and operating under their direct guidance. Secondly, LGFVs actively participate in the development and operation of local infrastructure projects. Thirdly, local governments inject financial resources and state-owned assets, such as land-use rights, into LGFVs to serve as collateral for fundraising in the financial markets. Lastly, LGFVs are guaranteed or implicitly committed by local governments. Therefore, the debt of LGFVs represents the implicit debt of local governments [22]. It is against this backdrop of rising LGFVs that we investigate the impact of LGD on firms’ labor income shares.

### 2.2 China’s banking-based financial system

The availability of bank lending plays a significant role in influencing firms’ business activities [23, 24]. Government debt financing introduces competition for funds between the government and corporate sectors, leading to a reduction in the flow of funds to businesses. In China’s current financial market landscape, direct financing is not the dominant mode, and the source of funds for enterprises mainly relies on bank loans, supplemented by corporate bonds for financing [25]. China’s financial system was initially established based on the banking system during the early stages of reform and opening up, with the stock market and bond market being relatively newer additions [26]. Compared to traditional bank financing, obtaining loans from banks is generally easier for enterprises, contributing to the underdevelopment of direct financing.

China’s credit market is fragmented along regional lines, with local banks facing restrictions when lending to firms located outside their cities. In terms of regulations, Article 59 of the General Rules for Loans issued by the People’s Bank of China governs inter-municipal loans for enterprises. This article stipulates that cross-city loans must be reported to the city branch of the People’s Bank of China, which increases the cost of cross-city loans. In practice, commercial banks not only contend with stricter external regulations for out-of-market loans but also face additional constraints from their internal risk control departments. Considering these factors, the cost for commercial banks to provide loans to firms located outside their cities is higher than the cost of providing loans to local firms, resulting in a geographical segmentation of China’s credit market [27].

Credit funds represent a crucial source of financing for firms, supporting labor hiring and payroll. Therefore, it is reasonable to hypothesize that LGFVs have a crowding-out effect on firms’ credit resources, consequently impacting firms’ labor income share.

## 3. Model construction and research hypothesis

### 3.1 Optimal production mix and labor income share

Borrowing from Klump et al. (2012), this paper constructs a theoretical framework to analyze how local government debt affects firms’ labor income shares [28]. Assuming that a firm uses two factors, capital K and labor L, to produce, the production function is *Y* = *Y*(K,L). From Euler’s theorem, it follows that.


Y=wL+rK (1)

Further, a firm’s share of labor income can be expressed in the following form:

$$LS=\frac{L\cdot Y_{L}}{Y}=\frac{wL}{Y}=\frac{wL}{wL+rK}=\frac{1}{1+(r/w)(K/L)}$$ (2)

In the above formula, LS represents labor share, while r and w represent capital and labor factor prices, respectively. To facilitate further derivation, this paper assumes that the enterprise’s production function is a constant elasticity of substitution (CES) function with invariant returns to scale, i.e.:

$$Y={A[\alpha K^{(\sigma -1)/\sigma }+(1-\alpha )L^{(\sigma -1)/\sigma }]}^{\sigma /(\sigma -1)}$$ (3)

Where *α*∈(0,1), *σ*∈[0,+∞) represent the substitution parameters, also known as factor elasticities of substitution. Moreover, A represents the technology coefficient, which characterizes the firm’s level of technology. The objective of a rational manufacturer is to choose between the optimal amount of capital and labor to be employed in order to maximize the firm’s profit, i.e.:

$${Max}_{K,L}\pi =Y-wL-rK$$ (4)

s.t.wL+rK≤C (5)

The firm chooses the optimal quantities of K and L through the profit maximization objective with the first-order condition that:

$$\frac{\partial \pi }{\partial K}=A\alpha K^{\frac{-1}{\sigma }}{\left[\alpha K^{\frac{\sigma -1}{\sigma }}+(1-\alpha )L^{\frac{\sigma -1}{\sigma }}\right]}^{\frac{1}{\sigma -1}}-r=0$$ (6)

$$\frac{\partial \pi }{\partial L}=A(1-\alpha )L^{\frac{-1}{\sigma }}{\left[\alpha K^{\frac{\sigma -1}{\sigma }}+(1-\alpha )L^{\frac{\sigma -1}{\sigma }}\right]}^{\frac{1}{\sigma -1}}-w=0$$ (7)

By shifting the terms of the above two equations, then dividing Eq (6) by Eq (7) and simplifying, we get:

$$\frac{r}{w}=(\frac{\alpha }{1-\alpha }){\left(\frac{L}{K}\right)}^{\frac{1}{\sigma }}$$ (8)

The proportion of capital and labor income in producer equilibrium can be derived by applying the appropriate operations to Eq (8):

$$\frac{rK}{wL}={\left(\frac{\alpha }{1-\alpha }\right)}^{\sigma }{\left(\frac{w}{r}\right)}^{\sigma -1}$$ (9)

Substituting Eq (9) into the far right-hand side of Eq (2) yields the firm’s share of labor income:

$$LS=\frac{1}{1+{\left(\frac{\alpha }{1-\alpha }\right)}^{\sigma }{\left(\frac{r}{w}\right)}^{1-\sigma }}$$ (10)

Further, by taking the partial derivative of the labor income share (LS) in Eq (10) for the capital factor price (r) and the labor factor price (w), respectively, the following two equations can be obtained:

$$\frac{\partial LS}{\partial r}=(\sigma -1)\frac{{\left(r/w\right)}^{1-\sigma }{\left[\alpha /(1-\alpha )\right]}^{\sigma }}{r{\left\{1+{\left(r/w\right)}^{1-\sigma }{\left[\alpha /(1-\alpha )\right]}^{\sigma }\right\}}^{2}}$$ (11)

$$\frac{\partial LS}{\partial w}=(1-\sigma )\frac{{\left(r/w\right)}^{1-\sigma }{\left[\alpha /(1-\alpha )\right]}^{\sigma }}{w{\left\{1+{\left(r/w\right)}^{1-\sigma }{\left[\alpha /(1-\alpha )\right]}^{\sigma }\right\}}^{2}}$$ (12)

According to Formulas (11) and (12), when capital factor price (r) or labor factor price (w) changes, the labor income share of enterprises will also change.

### 3.2 Consider the effect of local government debt

From Eq (11), we can see that LGD causes higher prices for firms’ capital factors, changing the labor income share. Excessive government demand for financing not only displaces credit resources originally available to enterprises but also indirectly raises their cost of financing [29]. Firstly, the overissuance of government debt tends to depress the price of treasury bonds while driving up their yield to maturity. The ‘‘asset portfolio effect” theory suggests that government debt and corporate debt, as distinct assets with differing risk profiles, compete for yields alongside other assets [30]. Financial institutions, acting as capital providers, adjust their asset portfolios by either increasing exposure to government debt or demanding higher returns from corporate borrowers. This shift inevitably leads to increased expenses associated with debt financing for corporations. Secondly, treasury bond yields are frequently employed as proxies for the risk-free rate of return in asset pricing models. As a result, when financial institutions extend loans to businesses, they often benchmark interest rates against government bond yields [31], thereby amplifying the cost of corporate debt financing. Thirdly, investments in government bonds, such as treasury bills, are typically perceived as safer than corporate bonds. The safety and liquidity associated with government bonds are akin to those of currencies, making them a preferred choice for investors over corporate bonds [32]. Consequently, an increase in LGD attracts investors who prioritize the safety of government securities. This, in turn, compels companies to offer higher bond yields to attract such investors, resulting in reduced debt financing for companies and higher associated costs [33]. Thus, an elevated level of government debt pushes up the price of corporate capital factors.

From Eq (12), it is clear that local government debt causes lower labor factor prices for firms, changing the labor income share. Local government debt exerts pressure on credit resources available to firms, thereby limiting their ability to secure working capital through credit channels. In a region, credit resources are finite, and within the competition for these resources between government and enterprises, banks often prioritize the financial needs of local governments. This preference results in a reduction in the availability of credit capital for businesses [34]. Given that local governments play a central role in regional economic development and exhibit substantial capital demands, the relatively stable money supply means that local government debt can displace enterprise debt financing, diminish bank loans to businesses, and intensify the competition between local government entities and enterprises for capital [35]. Firms typically require access to credit facilities from banks in their production processes, particularly for labor hiring [36]. Liquid cash is an important channel that affects the value of an enterprise, especially when external uncertainty occurs [37, 38]. Reduced access to credit, which subsequently reduces working capital, prompts firms to lower average wage levels, negatively impacting the share of labor income [39]. In response to financing challenges, firms are compelled to turn to internal financing methods, such as using profits or retaining cash, to address funding shortfalls. This, in turn, reduces the distribution of profits to labor income for the populace.

Eqs (11) and (12) show that local government debt can further lead firms to adjust their optimal factor allocations by changing the prices of production factors. Therefore, to see more intuitively the impact of relative factor price changes on the labor share, let $p=\frac{r}{w}$ be brought into Eq (10). Take the derivative of the labor income share (LS) concerning price (p):

$$\frac{dLS}{dp}=(\sigma -1)\frac{p^{-\sigma }{\left[\alpha /(1-\alpha )\right]}^{\sigma }}{{\left\{1+p^{1-\sigma }{\left[\alpha /(1-\alpha )\right]}^{\sigma }\right\}}^{2}}$$ (13)

Eq (13) shows that the changes in labor share due to changes in factor prices depend on an estimate of the elasticity of substitution. Therefore, we need to estimate it.

### 3.3 Capital labor factor elasticity of substitution

The elasticity of substitution is a critical variable affecting the effect of relative prices on the labor share, and estimating its value is one of the fundamental tasks in exploring the labor share of income [40, 41]. This paper uses the Taylor series expansion proposed by Kmenta (1967) to calculate the elasticity of substitution [42]. Taking logarithms for both the left and right sides of Eq (3), the following results are obtained:

$$lnY=lnA+\frac{\sigma }{\sigma -1}[\alpha K^{\frac{\sigma -1}{\sigma }}+(1-\alpha )L^{\frac{\sigma -1}{\sigma }}]$$ (14)

A Taylor polynomial expansion of Eq (14) at *σ* = 1 yield:

$$lnY=\beta_{0}+\beta_{1}lnK+\beta_{2}lnL+\beta_{3}{\left[ln\frac{K}{L}\right]}^{2}$$ (15)

where *β*_0_ = *lnA*, *β*_1_ = *α*, *β*_2_ = 1−*α* and $\beta_{3}=\frac{\sigma -1}{2\sigma }\alpha (1-\alpha )$. By estimating *β*_1_, *β*_2_ and *β*_3_, the capital and labor elasticities of substitution are obtained as follows:

$$\sigma =\frac{\beta_{1}\beta_{2}}{\beta_{1}\beta_{2}-2\beta_{3}(\beta_{1}{+\beta }_{2})}$$ (16)

In this paper, we estimate the elasticity of substitution in the sample by means of a two-way fixed effects model, and the elasticity of substitution is calculated to be 0.823, indicating that the elasticity of substitution for the firms is complementary. Therefore, we formulate the following hypothesis.

**Hypothesis**: Since capital and labor are complementary, local government debt reduces the firm’s share of labor income.

## 4. Empirical design

### 4.1 Data

We use Chinese listed companies as the research sample and select 2010–2022 as the sample interval. The specific sample data have been appropriately processed: (1) exclude companies in the financial sector; (2) exclude ST and *ST companies; (3) exclude companies with a total number of employees less than 100; (4) exclude samples with a labor income share more significant than one or less than zero; (5) exclude samples with missing variables and observations with abnormal values. As the linear regression model used in this paper is susceptible to outliers, this paper applies a before-and-after 1% quantile tailing process to all continuous variables. Further, we retrieve the corresponding LGFVs liability information through the WIND database and match them to the city level as a proxied variable for local government debt. Corporate financial characteristics data from the China Stock Market and Accounting Research (CSMAR) database. According to the above criteria, 12,848 valid observations were obtained in this paper. The data sources used in this paper are the public databases WIND (available at https://www.wind.com.cn/) and CSMAR databases (available at https://www.gtarsc.com/).

### 4.2 Definition of variables

#### 4.2.1 Labor income share (LS)

In this study, LS is expressed as ‘‘labor compensation/value added of the enterprise,” taking into account the existing literature [43]. Labor compensation is expressed as ‘‘cash paid to employees” in the cash flow table in the CSMAR database. Enterprise value added = operating income—operating costs + cash paid to and on behalf of employees + depreciation of fixed assets. In addition, in order to make the LS variable more consistent with normal distribution, further logarithmic processing of LS was carried out by referring to the practice of Yang and Tsou (2021) and expressed as ln(LS/(1-LS)) [44].

#### 4.2.2 Local government debt (LGD)

For the purposes of financing and promoting economic development, local governments have widely established state-owned investment companies, often referred to as local government financing vehicles (LGFVs). These entities represent local governments in infrastructure investments and have the ability to issue bonds or obtain loans from banks to raise funds. In assessing local government debt, we follow the approach proposed by Huang et al. (2020) and use the interest-bearing debt of LGFVs as a proxy variable for LGD [7]. This key explanatory variable, LGD, is expressed as the ‘‘interest-bearing debt of LGFVs as a percentage of GDP.” Interest-bearing debt of LGFVs includes bank borrowings, bond payable balances, notes payable, and non-current liabilities due within one year.

#### 4.2.3 Control variables (Controls)

With reference to Li et al. (2023) [45], the control variables mainly include enterprise size (Size), return on assets (ROA), enterprise age (Age), financial leverage (Lev), fixed asset size (Tang), enterprise value (Tobin), growth (Growth), capital intensity (Ci), shareholding concentration (Top1), proportion of independent directors (Indir), number of board members (Board), and management shareholding (Mhold) (see Table 1 for details).

**Table 1:** Variable definitions.

Variable Name	Abbreviations	Definitions
Explained variable	LS	Labor remuneration divided by enterprise value added
	ln(LS/(1-LS))	Logarithmic conversion of the labor income share
Explanatory variable	LGD	The ratio of interest-bearing debt of local government financing vehicles to GDP
Control variable	Size	The natural log of total assets
	Roa	Net profit divided by total assets
	Age	The natural logarithm of the listing years to the current period
	Lev	Total liabilities divided by total assets
	Tang	Net fixed assets divided by total assets
	Tobin	Market value of the business divided by total assets for the period
	Growth	Growth rate of operating income
	Ci	Total assets divided by operating revenues
	Top1	Expressed as a percentage of the shareholding of the largest shareholder
	Indir	Number of independent directors divided by the number of board members
	Board	Natural logarithm of board members
	Mhold	Number of shares held by management divided by the total number of shares

### 4.3 Model design and descriptive statistics

#### 4.3.1 Model design

In considering the effects of LGD on labor share, the following two-way fixed effects model is constructed concerning the existing literature [46]:

$${LS}_{i,t}=\beta_{0}+\beta_{1}\times {LGD}_{c,t}+\gamma \times {Controls}_{i,t}+\mu_{i}+\theta_{t}+\varepsilon_{i,t}$$ (17)

The subscript *i* denotes the firm, *t* denotes the year, and *c* denotes the city. *Controls* are a set of control variables. *μ*_*i*_ and *θ*_*t*_ denote fixed effects at the individual and time levels, respectively. Table 1 presents the specific variable definitions. Our study focuses on the coefficient of LGD (*β*_1_). If *β*_1_ is significantly verified as positive, this means that LGD positively impacts the labor share. On the contrary, if *β*_1_ is significantly verified as negative, this means that LGD has a negative impact on the labor share.

#### 4.3.2 Descriptive statistical analysis

Table 2 provides descriptive statistics for each variable. The dependent variable LS, for example, has a mean value of 0.281, indicating that the sample’s average labor income share is 28.1%. Meanwhile, the minimum value is 0.071, and the maximum value is 0.513, which shows a significant difference in the labor income share within the sample. The mean value of the independent variable, LGD, is 0.177, with a minimum value of 0 and a maximum value of 0.845. The statistical results of the other variables are also consistent with economic reality and will not be described in detail here.

**Table 2:** Descriptive statistics.

Variables	Num.	Mean	Std.	Min	p50	Max
LS	12848	0.281	0.101	0.071	0.277	0.513
ln(LS/(1-LS))	12848	-1.004	0.545	-2.573	-0.959	0.053
LGD	12848	0.177	0.182	0.000	0.130	0.845
Size	12848	22.266	1.224	19.973	22.110	26.047
Roa	12848	0.041	0.050	-0.153	0.036	0.192
Age	12848	0.281	0.036	0.161	0.289	0.343
Lev	12848	0.445	0.201	0.060	0.441	0.895
Tang	12848	0.385	0.171	0.037	0.375	0.790
Tobin	12848	0.202	0.122	0.089	0.161	0.783
Growth	12848	0.192	0.420	-0.436	0.117	2.885
Ci	12848	2.201	1.689	0.383	1.724	10.426
Top1	12848	0.355	0.152	0.086	0.337	0.762
Indir	12848	0.372	0.053	0.333	0.333	0.571
Board	12848	2.269	0.175	1.792	2.303	2.773
Mhold	12848	0.099	0.180	0.000	0.001	0.681

**Notes:** Variable definitions are provided in Table 1.

## 5. Results

### 5.1 Baseline regression

Table 3 provides the outcome of the benchmark regression. Column (1) presents the results of the univariate test of LGD on the labor share, where the coefficient of LGD is significantly negative. In addition, we use stepwise regression for the test, and the results in columns (2) and (3) show that the results remain significantly negative after the stepwise addition of the control variables. The results remain consistent in columns (4) to (6) when the dependent variable is ln(LS/(1-LS)). In summary of the results, it is clear that local government debt causes firms to reduce their share of corporate labor income.

**Table 3:** The impact of local government debt on the labor income share.

Variables	Dependent variable: LS			Dependent variable: ln(LS/(1-LS))		
	(1)	(2)	(3)	(4)	(5)	(6)
LGD	-0.030***	-0.028***	-0.031***	-0.157***	-0.149***	-0.164***
	(-2.92)	(-3.28)	(-3.64)	(-2.89)	(-3.25)	(-3.62)
Size		-0.026***	-0.030***		-0.147***	-0.166***
		(-18.47)	(-20.82)		(-19.62)	(-21.92)
Roa		-0.716***	-0.683***		-3.826***	-3.646***
		(-53.61)	(-50.45)		(-53.67)	(-50.47)
Age		0.080	-0.024		0.512	-0.067
		(1.03)	(-0.30)		(1.24)	(-0.16)
Lev		-0.006	-0.003		-0.029	-0.016
		(-1.07)	(-0.62)		(-1.03)	(-0.56)
Tang		-0.038***	-0.031***		-0.200***	-0.162***
		(-7.24)	(-5.89)		(-7.14)	(-5.77)
Tobin		-0.004	-0.006		-0.062*	-0.077**
		(-0.61)	(-1.02)		(-1.90)	(-2.33)
Growth		-0.012***	-0.009***		-0.067***	-0.054***
		(-10.33)	(-8.18)		(-10.95)	(-8.78)
Ci			0.006***			0.034***
			(11.25)			(11.06)
Top1			0.015*			0.039
			(1.81)			(0.91)
Indir			-0.027*			-0.146*
			(-1.75)			(-1.78)
Board			0.004			0.028
			(0.65)			(0.88)
Mhold			-0.063***			-0.324***
			(-8.76)			(-8.44)
Constant	0.286***	0.891***	0.984***	-0.976***	2.431***	2.926***
	(154.26)	(24.36)	(24.14)	(-98.18)	(12.45)	(13.44)
Firm fixed effects	Yes	Yes	Yes	Yes	Yes	Yes
Year fixed effects	Yes	Yes	Yes	Yes	Yes	Yes
Observations	12,848	12,848	12,848	12,848	12,848	12,848
R-squared	0.75	0.82	0.83	0.76	0.83	0.83

**Notes:** ***, ** and * are indicate significant levels of 1%, 5%, and 10% levels, respectively. T-statistics are provided in parentheses below each coefficient estimate.

### 5.2 Endogeneity tests

#### 5.2.1 DID model estimation

We employ exogenous event shocks to perform endogeneity tests. In response to the significant size expansion of LGFVs and the risk exposure of LGD in some cities, the central government’s policy stance towards LGFVs has transitioned from encouragement to stricter control in a relatively short timeframe [47, 48]. In 2014, the State Council issued ‘‘Opinions on Strengthening the Management of Local Government Debt,” often referred to as ‘‘Document 43.” The policy was drafted and promulgated independently by the central government and can, therefore, be seen as an excellent exogenous shock. The document stipulates that local government debt must be issued through government bonds. It suggests a clear separation of responsibilities between the government and enterprises, emphasizing that government debt should not be borrowed by enterprises and enterprise debt should not be repaid by the government. Furthermore, it limits the size of local government debt, which must not exceed the higher-level stipulation. Local government debt is categorized into two types: general debt and special debt. This classification weakens the financing role of LGFVs. The document aims to regulate the behavior of local government debt raising and restrain the disorderly expansion of LGFVs. From the preceding theoretical analysis, it becomes apparent that the governance of local debt can reduce the high-interest rate financing activities of LGFVs. This reduction not only decreases the cost of government borrowing and financing but also helps moderate competition in the market for fund demand, subsequently reducing market interest rates. Before the policy was enacted, a portion of corporate loans would flow into LGFVs and form loans from LGFVs. However, after the policy was enacted, this portion of local government loans significantly decreased. As a result, the available liquidity funds for firms increased, ultimately affecting their labor income share.

The policy has restricted the debt financing of local governments. Therefore, this paper refers to related scholars to treat high local government debt financing areas as a treatment group and low local government debt financing areas as the control group, using a DID model and the release of “Document 43” as an exogenous influence on local government debt financing, to explore whether this policy helps to alleviate the crowding out of the share of labor income of enterprises [49, 50]. We construct the following DID model:

$${LS}_{i,t}=\beta_{0}+\beta_{1}\times {Treat}_{i}\times {Post}_{t}+\gamma \times {Controls}_{i,t}+\mu_{i}+\theta_{t}+\varepsilon_{i,t}$$ (18)

We use the ex-ante (2010–2013) mean of the core explanatory variable LGD as a criterion for classifying the treatment group (*Treat* = 1) and the control group (*Treat* = 0). As expected, firms with ex-ante local government debt below the mean are less influenced by ‘‘Document 43”. They are classified in the control group (*Treat* = 0), while firms with ex-ante local government debt above the mean are more influenced by the policy document and are classified in the treatment group (*Treat* = 0). The dummy variable Post is used to measure policy shocks, with Post taking a value of 1 when the sample year is 2014 and later and 0 otherwise. The interaction term *Treat*_*i*_×*Post*_*t*_ represents the policy effect of the introduction of ‘‘Document 43” that we are interested in, which leads to changes in the labor income share of enterprises.

Table 4 shows the results of the estimates for the DID model to test endogeneity. The coefficient of the interaction term *Treat*_*i*_×*Post*_*t*_ can be found to be significantly positive. This indicates that the introduction of ‘‘Document 43” has reduced local government debt and alleviated the negative effect on the labor share. The empirical results based on quasi-natural experimental shocks reaffirm the hypothesis of this paper that local government debt causes a decrease in firms’ share of labor income; after local government debt financing is effectively managed, the labor income share of firms gradually increases.

**Table 4:** Quasi-natural impact test based on “Document 43”.

Variables	Dependent variable: LS		Dependent variable: ln(LS/(1-LS))	
	(1)	(2)	(3)	(4)
Treat×Post	0.009***	0.005***	0.043***	0.022**
	(4.24)	(2.93)	(3.82)	(2.36)
Constant	0.271***	0.972***	-1.040***	2.886***
	(52.91)	(23.63)	(-37.92)	(13.15)
Controls	No	Yes	No	Yes
Firm fixed effects	Yes	Yes	Yes	Yes
Year fixed effects	Yes	Yes	Yes	Yes
Observations	12,848	12,848	12,848	12,848
R-squared	0.75	0.83	0.76	0.83

**Notes:** ***, ** and * are indicate significant levels of 1%, 5%, and 10% levels, respectively. T-statistics are provided in parentheses below each coefficient estimate. Control variables include Size, Roa, Age, Lev, Tang, Tobin, Growth, Ci, Top1, Indir, Board, Mhold.

#### 5.2.2 Instrumental variable tests

This paper employs instrumental variables for endogeneity testing to mitigate possible endogeneity issues further. Concerning the research method of Demirci et al. (2019), we select health care expenditure in public financial expenditure as instrumental variables (IV1) [51]. The selection of instrumental variables should satisfy both relevance and exogeneity. The expenditure on health care is related to the revenue and expenditure of public finance, which is also related to the generation and change of local government debt. We consider health care expenditure as essential livelihood expenditure, relatively exogenous in government fiscal spending and less susceptible to exogenous factors. In addition, we take local government debt (IV2), which lags by one period, as the second instrumental variable.

Table 5 shows the regression results for the two instrumental variables. We used a two-stage approach to estimation. Columns (1) and (3) show the first-stage regression results for the two instrumental variables, respectively. The test results report that the correlation coefficients between the two instrumental and endogenous explanatory variables LGD are significantly positive. Moreover, the F-statistics of 28.53 and 2362.28 are much larger than the experience value of 10, ruling out the issue of weak instrumental variables. Columns (2) and (4) report the results of the second regression stage, with the coefficients of the key explanatory variables both significantly negative. The result suggests that higher levels of LGD, the lower the firms’ labor income share. After effectively controlling for possible endogeneity issues, the conclusions drawn from this study remain robust.

**Table 5:** Instrumental variable tests.

Variables	LGD	LS	LGD	LS
	IV1 First Stage	IV1 Second Stage	IV2 First Stage	IV2 Second Stage
	(1)	(2)	(3)	(4)
IV1	0.040***			
	(5.34)			
IV2			0.425***	
			(48.60)	
LGD		-1.827**		-0.043**
		(-2.07)		(-2.18)
Controls	Yes	Yes	Yes	Yes
Firm fixed effects	Yes	Yes	Yes	Yes
Year fixed effects	Yes	Yes	Yes	Yes
Observations	12848	12848	12848	12848
F-Statistic	28.53		2362.28	
p-value	0.000		0.000	

**Notes:** ***, ** and * are indicate significant levels of 1%, 5%, and 10% levels, respectively. T-statistics are provided in parentheses below each coefficient estimate. Control variables include Size, Roa, Age, Lev, Tang, Tobin, Growth, Ci, Top1, Indir, Board, Mhold.

### 5.3 Robustness test

#### 5.3.1 Parallel trend test

The regression results mentioned above may be biased if before the implementation of the LGD governance policy, the labor income shares of the treatment and control groups show different trends. Therefore, when employing the DID model, it is crucial for the treatment and control groups to fulfill the parallel trend assumption. Moreover, the regression results based on Eq (18) only analyze the changes in the average labor share before and after the implementation of the LGD governance policy without considering the dynamic impact of policy implementation on the labor share each year. To address this, we follow the methodology outlined by Chen et al. (2021) and utilize an event study approach to assess ex-ante parallel trends and examine ex-post marginal effects across years [52]. The following extended DID dynamic effects model is constructed based on Eq (18):

$${LS}_{i,t}=\beta_{0}+\beta_{k}\times \sum_{k=2010,k\neq 2013}^{k=2018}{Year}_{k}{\times Treat}_{i}+\gamma \times {Controls}_{i,t}+\mu_{i}+\theta_{t}+\varepsilon_{i,t}$$ (19)

Using 2013, the year before the policy occurred, as the base year, *Year*_*k*_ denotes a series of year dummy variables for 2010–2018. The parameter *β*_*k*_ identifies the impact of the policy implementation before (or after) it occurs in year k. If *β*_2010_−*β*_2012_ are not significantly different from 0, it indicates that the parallel trend is satisfied.

**Fig 1:**
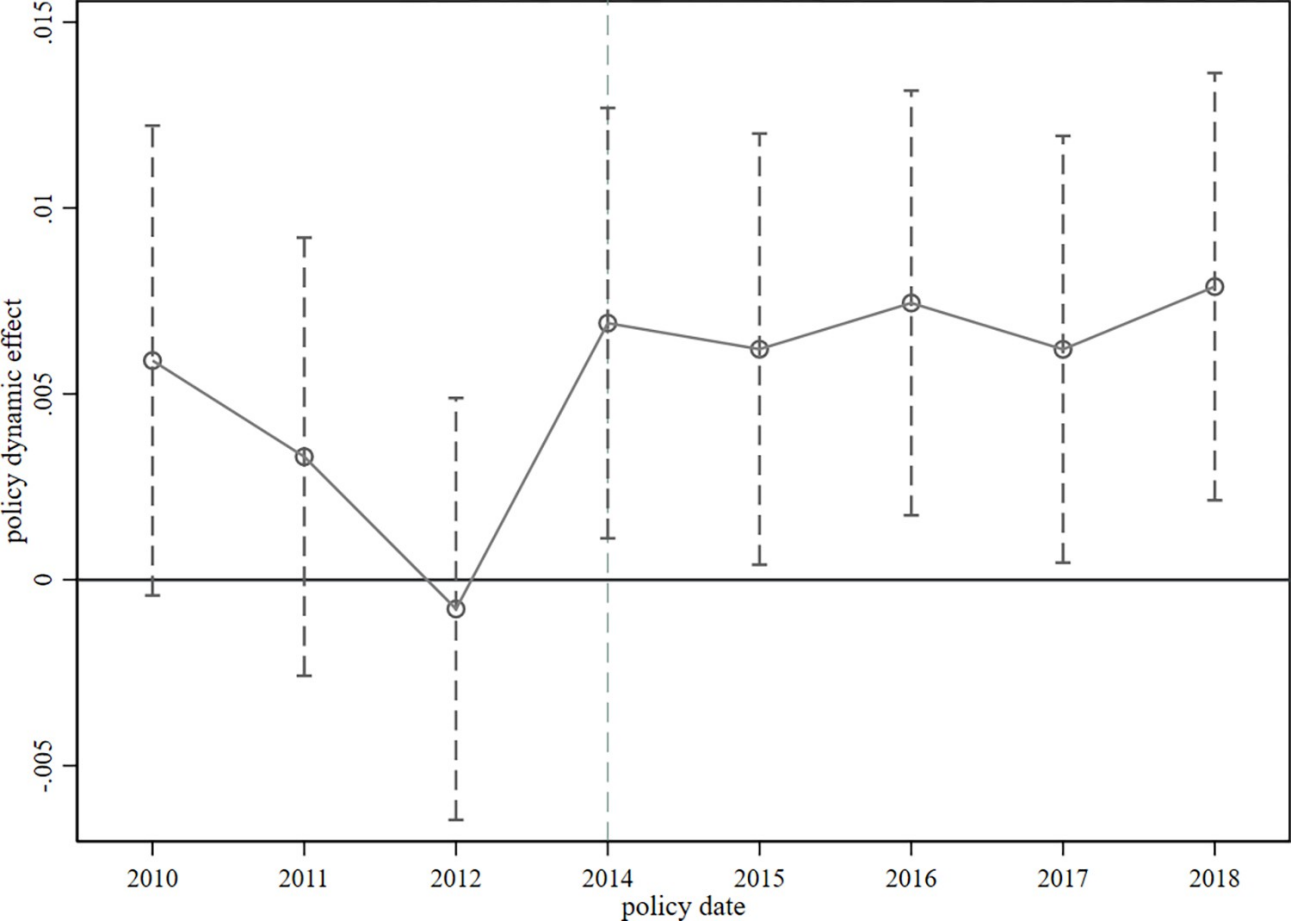
Parallel trend test.

Fig 1 distinctly illustrates the causal relationship between LGD and labor share over the time series. The dots in the figure indicate the *β*_*k*_ estimates, and the dashed lines through the dots and perpendicular to the x-axis indicate the 95% horizontal confidence intervals. The coefficients of *Year*_2010_×*Treat*−*Year*_2012_×*Treat* are all statistically insignificant, implying that prior to the implementation of the local government debt governance policy, there is no significant disparity in labor share between the treatment group and the control group, thereby satisfying the parallel trend assumption. Conversely, the coefficients of *Year*_2014_×*Treat*−*Year*_2018_×*Treat* are all significantly positive, signifying that the implementation of governance policies leads to an increase in labor share.

#### 5.3.2 Modifying the model settings

Table 6 provides the regression results after replacing the fixed effects model. In this case, we have substituted fixed effects with firm, year, city, and industry fixed effects, as per the fixed effects model. Column (1) illustrates the outcomes of regressing LGD on labor income share after including city-fixed effects in the baseline model. Column (2) demonstrates the findings of regressing LGD on labor income share after introducing city- and industry-fixed effects into the baseline model. Columns (3) and (4) represent the regression outcomes when the dependent variable is altered to ln(LS/(1-LS)). The results show that the coefficient of the primary explanatory variable (LGD) remains negative, aligning with the outcomes of the benchmark regression. Consequently, the conclusions drawn in this study remain robust and substantiated.

**Table 6:** Modifying the model settings.

Variables	Dependent variable: LS		Dependent variable: ln(LS/(1-LS))	
	(1)	(2)	(3)	(4)
LGD	-0.031***	-0.031***	-0.168***	-0.165***
	(-3.68)	(-3.67)	(-3.72)	(-3.72)
Constant	0.995***	0.953***	2.971***	2.720***
	(24.36)	(23.58)	(13.65)	(12.66)
Controls	Yes	Yes	Yes	Yes
Firm fixed effects	Yes	Yes	Yes	Yes
Year fixed effects	Yes	Yes	Yes	Yes
City fixed effects	Yes	Yes	Yes	Yes
Industry fixed effects	No	Yes	No	Yes
Observations	12,848	12,848	12,848	12,848
R-squared	0.83	0.84	0.84	0.84

**Notes:** ***, ** and * are indicate significant levels of 1%, 5%, and 10% levels, respectively. T-statistics are provided in parentheses below each coefficient estimate. Control variables include Size, Roa, Age, Lev, Tang, Tobin, Growth, Ci, Top1, Indir, Board, Mhold.

#### 5.3.3 Replacing the dependent variable

Table 7 shows the regression results of replacing the dependent variables. In 2007, China issued new accounting standards, and listed companies must disclose detailed employee compensation changes in their financial statement notes. This paper selects the credit amount of employee compensation under ‘‘compensation payable” as the second proxy index of labor compensation (LS2). Column (1) displays the results of the univariate regression of LGD on LS2. Column (2) presents the results of the regression after adding the control variables. Columns (3) and (4) showcase the regression outcomes when the dependent variable is transformed into ln(LS2/(1-LS2)). Notably, the results consistently show that the coefficient of the primary explanatory variable (LGD) remains negative, irrespective of controlling for the control variables or not. This alignment with the benchmark regression results reaffirms the validity of the findings in this study.

**Table 7:** Replacing the dependent variable.

Variables	Dependent variable: LS2		Dependent variable: ln(LS2/(1-LS2))	
	(1)	(2)	(3)	(4)
LGD	-0.021**	-0.022***	-0.109**	-0.114**
	(-2.07)	(-2.59)	(-1.99)	(-2.51)
Constant	0.277***	0.977***	-1.026***	2.900***
	(151.16)	(24.41)	(-102.53)	(13.33)
Controls	No	Yes	No	Yes
Firm fixed effects	Yes	Yes	Yes	Yes
Year fixed effects	Yes	Yes	Yes	Yes
Observations	12,848	12,848	12,848	12,848
R-squared	0.75	0.83	0.76	0.84

**Notes:** ***, ** and * are indicate significant levels of 1%, 5%, and 10% levels, respectively. T-statistics are provided in parentheses below each coefficient estimate. Control variables include Size, Roa, Age, Lev, Tang, Tobin, Growth, Ci, Top1, Indir, Board, Mhold.

#### 5.3.4 Excluding four municipalities directly under the central government

Table 8 investigates the influence of LGD on firms’ labor income shares by excluding the sample of four municipalities. These four cities, namely Beijing, Tianjin, Shanghai, and Chongqing, hold special political significance in China, and differences in the behavioral patterns of various levels of government in these cities may impact the business activities of firms in the region. Consequently, we excluded firms in these four cities from the analysis. Column (1) presents the results of the univariate regression of LGD on LS. Column (2) shows the regression results after adding the control variables. Columns (3) and (4) showcase the regression outcomes when the dependent variable is transformed into ln(LS/(1-LS)). Notably, the results consistently indicate that the coefficient of LGD remains negative with or without controlling for the control variables. This alignment with the results of the benchmark regression corroborates the robustness and validity of the findings in the present study.

**Table 8:** Excluding four municipalities directly under the central government.

Variables	Dependent variable: LS		Dependent variable: ln(LS/(1-LS))	
	(1)	(2)	(3)	(4)
LGD	-0.022**	-0.028***	-0.112*	-0.143***
	(-2.05)	(-3.09)	(-1.93)	(-2.99)
Constant	0.282***	0.980***	-0.998***	2.913***
	(147.14)	(22.01)	(-96.74)	(12.21)
Controls	No	Yes	No	Yes
Firm fixed effects	Yes	Yes	Yes	Yes
Year fixed effects	Yes	Yes	Yes	Yes
Observations	10,489	10,489	10,489	10,489
R-squared	0.75	0.83	0.75	0.83

**Notes:** ***, ** and * are indicate significant levels of 1%, 5%, and 10% levels, respectively. T-statistics are provided in parentheses below each coefficient estimate. Control variables include Size, Roa, Age, Lev, Tang, Tobin, Growth, Ci, Top1, Indir, Board, Mhold.

#### 5.3.5 Placebo tests

**Fig 2:**
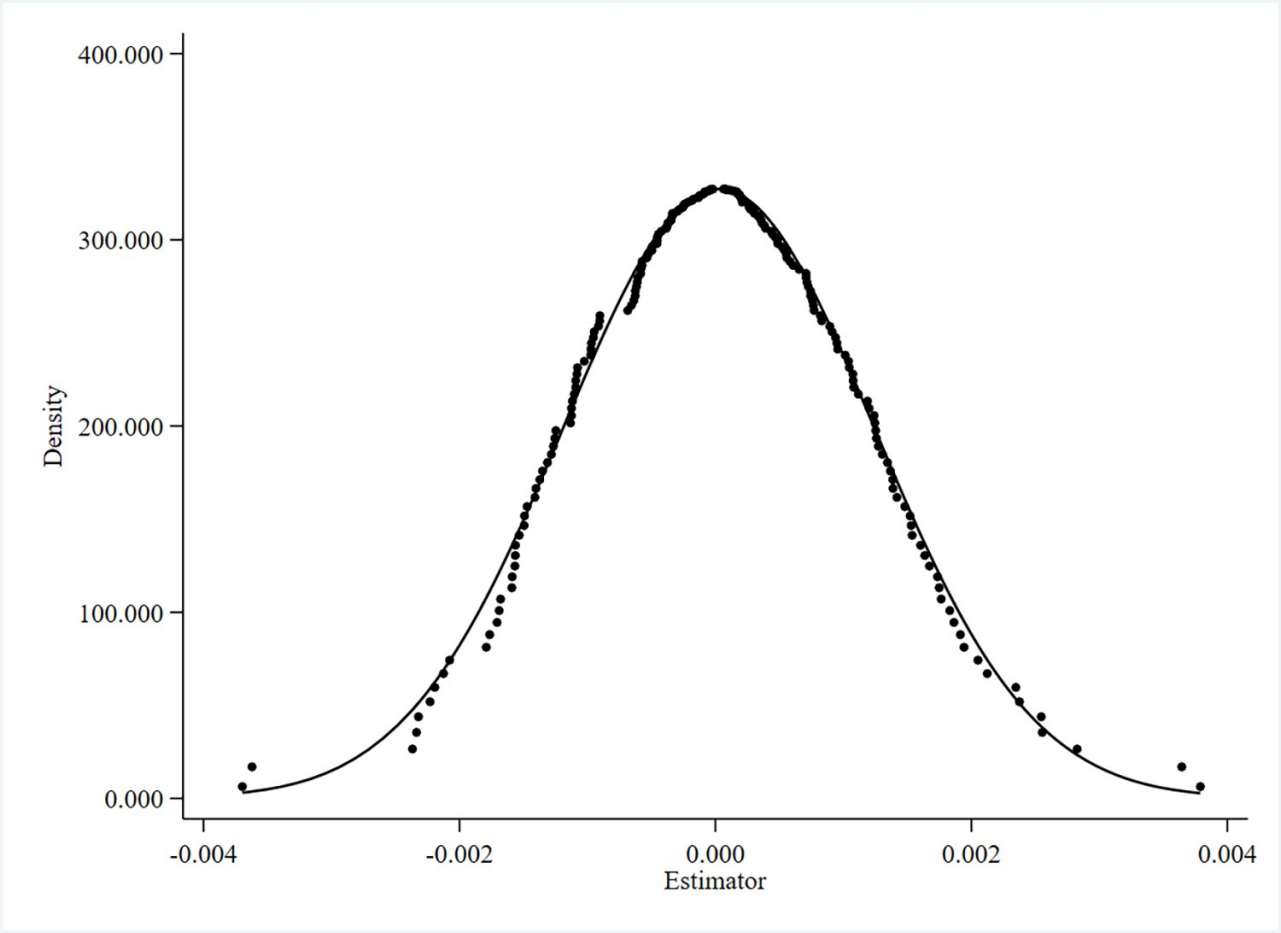
Random placebo test.

Fig 2 presents the first placebo test results. The first placebo test involved the random assignment of treatment groups. This was done to assess whether the observed policy effect could be attributed to other random factors. The aim was to eliminate any potential economic consequences stemming from these random factors, thereby achieving a more credible causal identification of the policy effect [53, 54]. By randomly selecting the treatment groups, we performed 200 replications and plotted the estimated distribution of *Treat*_*i*_×*Post*_*t*_ coefficients. We can find that the estimated coefficients of the spurious DID term are mainly concentrated near zero, which indicates that there is no significant omitted variable problem, and therefore the findings are still robust.

Table 9 presents the second placebo test results. Specifically, to confirm that local government debt governance policies caused a change in firms’ labor income shares and to remove the confounding effect of other random factors, we set a pseudo-policy time by advancing the policy time by one and two years and ran regressions using a DID model. Columns (1) and (2) are the results of regressions that advance the policy by one year; columns (3) and (4) are the results of regressions that advance the policy by two years. None of the coefficients from the regressions are significant for the DID regression model terms constructed through spurious policy timing.

**Table 9:** Fictitious policy times.

Variables	One year ahead		Two years ahead	
	LS	ln(LS/(1-LS))	LS	ln(LS/(1-LS))
	(1)	(2)	(3)	(4)
Treat×Post	0.002	0.004	0.003	0.010
	(1.12)	(0.37)	(1.50)	(0.86)
Constant	0.963***	2.811***	0.974***	2.900***
	(23.16)	(12.66)	(23.70)	(13.21)
Controls	Yes	Yes	Yes	Yes
Firm fixed effects	Yes	Yes	Yes	Yes
Year fixed effects	Yes	Yes	Yes	Yes
Observations	12,848	12,848	12,848	12,848
R-squared	0.83	0.83	0.83	0.83

**Notes:** ***, ** and * are indicate significant levels of 1%, 5%, and 10% levels, respectively. T-statistics are provided in parentheses below each coefficient estimate. Control variables include Size, Roa, Age, Lev, Tang, Tobin, Growth, Ci, Top1, Indir, Board, Mhold.

## 6. Further analysis

### 6.1 Mechanism analysis

The empirical analysis in the previous section argues that local government debt reduces the labor share. What is the mechanism by which this effect arises? Further, this paper tests how local government debt affects labor income shares. For a more intuitive analysis, we refer to Luo (2020) to formulate the labor income share as [55]:

$$LS=\frac{wL}{Y}=\frac{w}{Y/L}$$ (20)

Taking the logarithmic operation on Eq (20):

lnLS=lnw−ln(Y/L) (21)

Where w denotes the average wage rate (wages per capita), and *Y*/*L* denotes labor productivity (value added per capita). Eq (21) shows that the extent to which local government debt causes a decrease in the labor income share of firms varies, depending on the direction and magnitude of changes in *lnw* and *ln* (*Y*/*L*).

Table 10 reports the regression results for the mechanism analysis. As can be seen from column (1), after taking the logarithm, local government debt still decreases the labor share. From columns (2) and (3), the regression coefficients are significantly negative for the average wage rate and negative but insignificant for labor productivity per capita. This indicates that local government debt affects the share of labor income of enterprises mainly through average wages. From column (4), it is clear that local government debt significantly reduces firm labor hiring, a result that is similar to Acharya et al. (2018) [56]. The conclusion aligns with the theoretical analysis presented in the preceding section. Firms often require borrowing to sustain labor hiring and maintain production operations. When borrowing is constrained, it results in reduced working capital, compelling firms to depend on internal financing sources like profits or retained cash to bridge the funding gap. This, in turn, leads to workforce reductions and decreased labor hiring by firms. In China’s financial markets, direct financing is not dominant, and firms usually depend primarily on bank loans with corporate bonds as a secondary means of financing. Based on the survey, 50% of government debt comes from bank loans, indicating that local government debt relies mainly on bank loans. Columns (5) and (6) show that LGD increases the cost of loans and squeezes out credit resources from firms. When considering their asset portfolios, financial institutions face two choices: either increase their allocation to government debt or demand a higher rate of return on firms’ debt. In either case, this leads to an increase in the cost of debt financing for firms. Financial institutions often use government bond yields as a benchmark for setting loan interest rates. This practice further elevates the cost of debt financing for firms. Government bonds are considered safer and more liquid investments, making them more attractive to investors. As a result, the surge in local government debt issuance attracts investors who are less inclined to hold corporate bonds. This, in turn, compels firms to raise bond yields to attract these investors, thereby increasing the cost of debt financing for firms and driving up the cost of corporate capital.

**Table 10:** The mechanism analysis.

Variables	lnLS	lnw	ln(Y/L)	lnL	Cost	Loan
	(1)	(2)	(3)	(4)	(5)	(6)
LGD	-0.119***	-0.131***	-0.036	-0.237***	0.075**	-0.012**
	(-3.51)	(-3.11)	(-1.05)	(-3.76)	(2.08)	(-2.13)
Constant	1.592***	1.706***	-0.022	-8.159***	-0.444**	-43.755***
	(9.78)	(8.41)	(-0.13)	(-26.99)	(-2.57)	(-11.70)
Controls	Yes	Yes	Yes	Yes	Yes	Yes
Firm fixed effects	Yes	Yes	Yes	Yes	Yes	Yes
Year fixed effects	Yes	Yes	Yes	Yes	Yes	Yes
Observations	12,848	12,848	12,848	12,848	12,848	12,848
R-squared	0.83	0.97	0.98	0.93	0.62	0.73

**Notes:** ***, ** and * are indicate significant levels of 1%, 5%, and 10% levels, respectively. T-statistics are provided in parentheses below each coefficient estimate. Control variables include Size, Roa, Age, Lev, Tang, Tobin, Growth, Ci, Top1, Indir, Board, Mhold.

### 6.2 Heterogeneity analysis

#### 6.2.1 Enterprise ownership heterogeneity

Table 11 shows a sub-sample discussion from the perspective of the nature of ownership. In the Chinese institutional context, state-owned enterprises (SOEs) have a natural ‘‘blood” relationship with the government, which has an apparent ‘‘paternalistic effect” on them, enabling state-controlled listed companies to obtain financial support such as bank loans at a lower cost [57]. The crowding out of corporate credit facilities by LGD is mainly found in non-state-owned enterprises, while state-owned enterprises are unaffected [7]. As a result, private firms prefer to use internal financing to alleviate external financing constraints. This will lead to different responses to labor income shares by firms with different ownership properties. According to the sub-sample regression results, the impact of LGD on firms’ labor income share is only significant in non-SOEs but not in SOEs.

**Table 11:** Enterprise ownership heterogeneity.

Variables	Dependent variable: LS		Dependent variable: ln(LS/(1-LS))	
	SOEs	Non-SOEs	SOEs	Non-SOEs
	(1)	(2)	(3)	(4)
LGD	-0.017	-0.048***	-0.093	-0.238***
	(-1.56)	(-3.54)	(-1.59)	(-3.48)
Constant	1.019***	0.806***	3.321***	1.712***
	(19.44)	(11.48)	(11.46)	(4.79)
Controls	Yes	Yes	Yes	Yes
Firm fixed effects	Yes	Yes	Yes	Yes
Year fixed effects	Yes	Yes	Yes	Yes
Observations	7,067	5,781	7,067	5,781
R-squared	0.83	0.84	0.83	0.85

**Notes:** ***, ** and * are indicate significant levels of 1%, 5%, and 10% levels, respectively. T-statistics are provided in parentheses below each coefficient estimate. Control variables include Size, Roa, Age, Lev, Tang, Tobin, Growth, Ci, Top1, Indir, Board, Mhold.

#### 6.2.2 Enterprise size heterogeneity

In general, enterprises of different sizes command different resource endowments. In China’s market economy system, the differences in resource conditions between large and small enterprises may be further magnified by the government’s preference for large enterprises. In credit markets, Chinese banks often prefer to extend loans to large enterprises, and as a result, large enterprises may encounter fewer financing constraints [58]. This has resulted in credit resources, dominated by state-owned banks, mainly flowing to large enterprises with ample capital, creating a rejection of small and medium enterprises. As a result, the labor share of different sized firms may have different characteristic manifestations. Referring to Bronzini and Iachini (2014), this paper generates a dummy variable for firm size by selecting the median of the total assets of firms, divides the sample of firms into large-scale and small-scale firms, and tests for firm size heterogeneity [59].

Table 12 reports the regression results for firm size heterogeneity. The results show that the significance levels of LGD regression coefficients differ for firms of different sizes. The regression coefficient of LGD is significantly negative for small-scale enterprises. In contrast, the regression coefficients for large-scale enterprises are statistically insignificant.

**Table 12:** Enterprise size heterogeneity.

Variables	Dependent variable: LS		Dependent variable: ln(LS/(1-LS))	
	Large-scale	Small-scale	Large-scale	Small-scale
	(1)	(2)	(3)	(4)
LGD	-0.013	-0.035**	-0.062	-0.228***
	(-1.11)	(-2.29)	(-0.97)	(-2.76)
Constant	0.717***	1.447***	1.526***	5.300***
	(8.70)	(19.94)	(3.46)	(13.45)
Controls	Yes	Yes	Yes	Yes
Firm fixed effects	Yes	Yes	Yes	Yes
Year fixed effects	Yes	Yes	Yes	Yes
Observations	6,513	6,335	6,513	6,335
R-squared	0.89	0.90	0.90	0.91

**Notes:** ***, ** and * are indicate significant levels of 1%, 5%, and 10% levels, respectively. T-statistics are provided in parentheses below each coefficient estimate. Control variables include Size, Roa, Age, Lev, Tang, Tobin, Growth, Ci, Top1, Indir, Board, Mhold.

#### 6.2.3 Heterogeneity of financing constraints

The theoretical analysis suggests that the scale of LGD expansion exacerbates firms’ external financing constraints and causes changes in firms’ factor prices, thereby reducing the share of labor income. Therefore, enterprises with varying degrees of financing constraints will exhibit different behaviors. We measure the degree of financing constraints of firms in two ways: by referring to the SA financing constraint indicator constructed by Hadlock and Pierce (2010) and by classifying firms into low and high financing constraint groups [60].

Table 13 shows the test results. The regression coefficients in column (1) for firms with high financing constraints are negatively significant; in column (2) for enterprises with low financing constraints, the regression coefficients are insignificant. Regression findings suggest the greater impact of local debt on firms with higher financing constraints. In contrast, firms with non-financing or low financing constraints are unaffected. It is clear from the regression results that LGD has a more significant effect on enterprises with higher financing constraints. The results remain consistent when the dependent variable is ln(LS/(1-LS)).

**Table 13:** Financing constraint heterogeneity.

Variables	Dependent variable: LS		Dependent variable: ln(LS/(1-LS))	
	High financing constraints	Low financing constraints	High financing constraints	Low financing constraints
	(1)	(2)	(3)	(4)
LGD	-0.028*	-0.013	-0.208**	-0.046
	(-1.67)	(-1.01)	(-2.32)	(-0.69)
Constant	1.022***	0.970***	2.914***	2.996***
	(14.37)	(10.74)	(7.69)	(6.32)
Controls	Yes	Yes	Yes	Yes
Firm fixed effects	Yes	Yes	Yes	Yes
Year fixed effects	Yes	Yes	Yes	Yes
Observations	6,439	6,409	6,439	6,409
R-squared	0.91	0.89	0.91	0.90

**Notes:** ***, ** and * are indicate significant levels of 1%, 5%, and 10% levels, respectively. T-statistics are provided in parentheses below each coefficient estimate. Control variables include Size, Roa, Age, Lev, Tang, Tobin, Growth, Ci, Top1, Indir, Board, Mhold.

## 7. Conclusion and recommendations

Increasing the share of people’s labor income is a critical way to ensure that residents share the fruits of economic development. Therefore, exploring the factors influencing labor income share is of great theoretical and practical significance for achieving common prosperity. Chinese local government debt has expanded recently, and its risks and consequences still need to be fully understood. Unfortunately, no literature has been conducted to examine how government debt affects firms’ share of labor income. This paper adds to this aspect. The study finds that (1) local government debt financing significantly suppresses firms’ labor income share. The results pass the endogeneity test and detailed robustness tests. (2) The mechanism of action is that LGD significantly reduces firms’ per capita pay, labor demand, and bank loans. In addition, local governments significantly increase the cost of using capital for firms, further altering the price of capital-labor factors for firms. (3) Heterogeneity tests found that the dampening effect of LGD was more significant in non-state enterprises, SMEs and enterprises with high financing constraints.

Our empirical findings provide some policy implications. First, enhance regulations pertaining to local government debt management. Countries with more established practices in local government debt management have developed effective legal frameworks to ensure that local governments raise debts legally, utilize them according to the law, repay them systematically, and maintain controlled risks. Local governments compensate for the funding gap required for local economic development and infrastructure projects by establishing local investment and financing platforms with governmental affiliations. This has led to a rapid expansion of local government debt. Furthermore, most methods for managing local government debt are formulated by local governments themselves, which, compared to the national debt management system, are often incomplete and lacking in systemization, resulting in weak constraints. Second, local government debt should be integrated into budget management. Apart from local government bonds and various financial transfer funds, a significant portion of local government debt revenue and expenditure must still be included in budget management and oversight. This detachment from budget constraints has led to disorderly management. Inadequate supervision of local government debt ultimately means that it, from its source to its utilization and repayment, falls outside the scope of budgeting and, consequently, beyond the purview of higher-level government bodies. Third, raise awareness regarding the management of local government debt risks. Debt is a double-edged sword. While local government debt plays a constructive role in addressing funding shortfalls for local economic development, crisis response, improvement of living standards, environmental protection, and the promotion of sustainable local economic and social development, its continuous expansion has given rise to several issues. An extensive accumulation of bank loans may trigger liquidity risks for financial institutions. Furthermore, servicing the debt may further exacerbate local governments’ reliance on land-based financing.

Further research can be undertaken in the following directions. First, appropriate government debt can promote economic development and improve people’s livelihood. Excessive government debt, on the other hand, will accumulate substantial debt risks. Therefore, finding the proper debt equilibrium is what we need to further study in the future. Second, social welfare includes many aspects, and corporate labor income is only one. The welfare effect of local government debt on society can be further analyzed. Third, this study examines the share of labor income from the perspective of enterprises and whether it is possible to study it from the residents’ perspective.
